# Response: Commentary: Control of Body Weight by Eating Behavior in Children

**DOI:** 10.3389/fped.2016.00017

**Published:** 2016-03-10

**Authors:** Per Södersten, Modjtaba Zandian, Cecilia E. K. Bergh

**Affiliations:** ^1^Karolinska Institutet, Huddinge, Sweden

**Keywords:** childhood obesity, eating behavior, external control, internal control, body weight

**To the Editor:**

We thank Dr. Tony Kuo for his valuable comments on our review. As Dr. Kuo correctly points out, interventions aiming at manipulating internal mechanisms related to body weight, e.g., ­pharmacology and diets, have had limited success in reversing the increase in pediatric body weight. We agree with him that the alternative suggestion that body weight is under external control is in line with many recent public health initiatives aiming at improving the food environment. Dr. Kuo listed many of the newer policies, some of which we were unaware of and so we learnt a great deal from reading Dr. Kuo’s commentary paper. Yet, we feel confident that Dr. Kuo agrees with us that the success of these initiatives in curbing further increase in body weight among children has also been limited.

We beg to disagree, however, with Dr. Kuo that the hypothesis that body weight is under external control is “opposed to the viewpoint that cognition plays a predominant role,” and that our method is somehow related to “cognitive control.” We refer to the compelling work of Dr. Brian Wansink, showing that the role of cognition in eating behavior is negligible (1). Undesirable cognitions, including “dietary restraint,” among obese individuals are more likely the results of failed attempts at dieting than causes of their weight problem (2). Interestingly, a sustained reduction of body weight has been obtained with cognitive behavioral therapy (3), but it is clear from the protocol that the effect was obtained by behavioral intervention, i.e., establishing normal eating behavior; whatever effect the cognitive intervention exerted was less important (4).

At the same time, we certainly agree with Dr. Kuo that our suggestion that body weight can increase among the underweight and decrease among the overweight if they learn how to eat normally by using feedback on a computer screen requires further investigation. However, the facts that this method has brought 75% of patients with eating disorders into remission, reduced the rate of relapse to 10% over 5 years of follow-up, and eliminated the mortality are encouraging (5). We have outlined the neurobiological mechanisms on which the method relies in detail (references in our review). The approach was adapted to the treatment of childhood obesity because anorexia and obesity are at the extreme opposites of the same behavioral and biochemical continuum (6). Dr. Kuo is right in suggesting that the success of this approach on a larger scale needs to be tested. And, of course, creating healthy environments, including advice on diets and physical activity, remains important not least “for those who live in low socioeconomic status areas.”

## Author Contributions

All authors worked on the response and all agreed on the final version of the manuscript.

## Conflict of Interest Statement

PS and CB own 47.5% each of the stock in Mando Group AB; Michael Leon owns 5%. Mando Group AB holds the IPR of Mandometer, the FDA-approved medical device used to treat patients with eating disorders in clinics managed by Mando Group AB. Swedish Healthcare is publically funded. MZ and PS are appointed by Karolinska Institutet; all salaries are paid by Mando Group AB.
